# Supplementary material for: Comparison of Antibody Responses against Two Molecules from Ascaris lumbricoides: The Allergen Asc l 5 and the Immunomodulatory Protein Al-CPI
Source: Biology (Basel). 2023 Oct 16;12(10):1340. doi: 10.3390/biology12101340 (PMC10604738; doi:10.3390/biology12101340)
Supplement: Supplementary file 1 [file biology-12-01340-s001.zip › biology-2592512-supplementary.pdf]

Table S1. Dilutions for ELISA assays in mouse experiments.

Isotypes	Plasma dilutions	Secondary antibody dilutions
IgE	1:10	1:1000
IgG1	1:80000	1:10000
IgG2a	1:320	1:1000

Table S2. Relationship between antibody responses to Al-CPI and Asc l 5 with Ascaris infection.

	Non-infected		Infected	
rAsc l 5				
IgE+	52	54.7	99	50.8
IgG+	62	67.4	139	72.0
IgG4+	68	73.9	128	66.3
rAl-CPI				
IgE+	42	44.2	77	39.5
IgG+	65	70.7	150	77.7
IgG4+	73	79.3	158	81.4

Table S3. Median levels of antibody responses to Ascaris antigens.

Antigen	Non-infected			Infected		
	Median	IQR		Median	IQR	
		25	75		25	75
**rAsc l 5**						
IgE+	0,157	0,11	0,26	0,15	0,12	0,22
IgG+	0,363	0,31	0,42	0,39	0,32	0,46
IgG4+	0,217	0,15	0,55	0,20	0,16	0,32
**rAl-CPI**						
IgE+	0,124	0,10	0,19	0,12	0,10	0,18
IgG+	0,407	0,29	0,54	0,44	0,33	0,55
IgG4+	0,258	0,17	0,48	0,25	0,18	0,44

IQR: interquartile range.

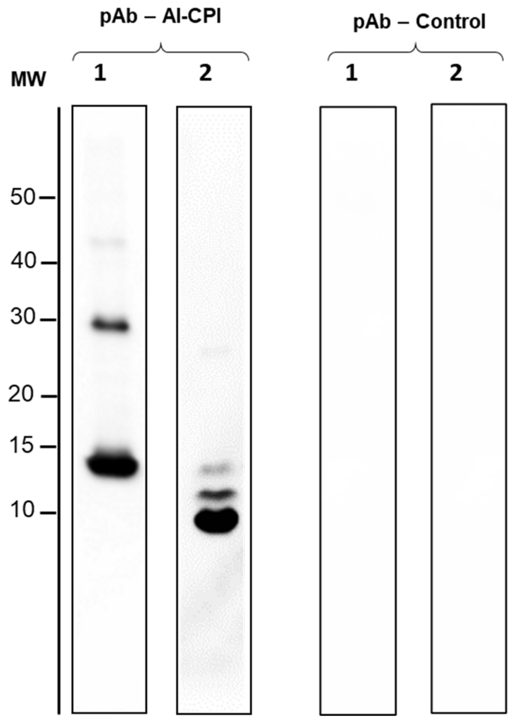

Figure S1. Recognition of CPI in the natural extract of *Ascaris lumbricoides*. Left, Anti-Al-CPI polyclonal antibody raised from three rAl-CPI immunized mice (pAb-Al-CPI) was tested against rAl-CPI (Lane 1) or the natural extract of *A. lumbricoides* (Lane 2). Right, same experiments were performed with PBS-immunized mice. Difference in the molecular weight (MW) between native and recombinant Al-CPI is due to the extra amino acids added in the recombinant plasmid.

A

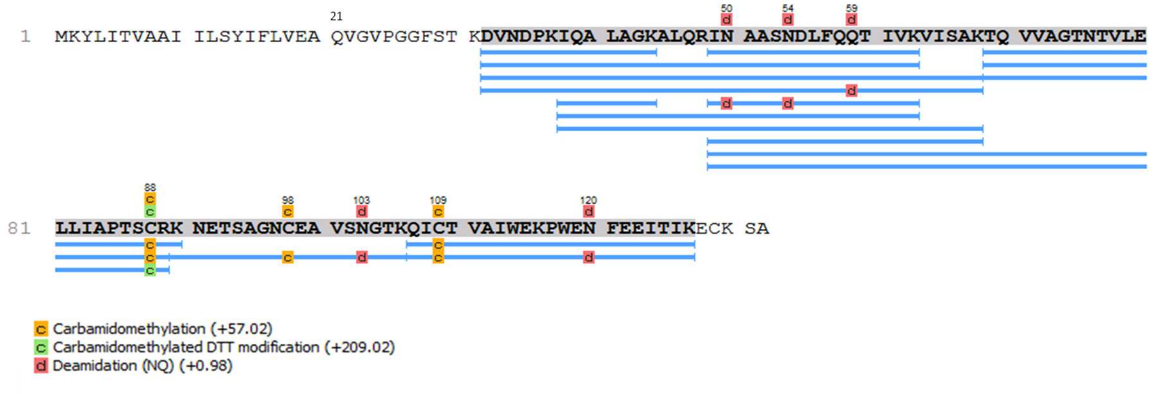

Figure S2. Mass spectrometric analysis of peptide coverage of natural Al-CPI. Blue lines indicate peptides in the *A. lumbricoides* extract matching mature sequence of Al-CPI. The amino acid sequence of Al-CPI (HQ404231.1) is shown. Amino acids from 1 to 20 correspond to the signal peptide. Mature Al-CPI starts in amino acid number 21.

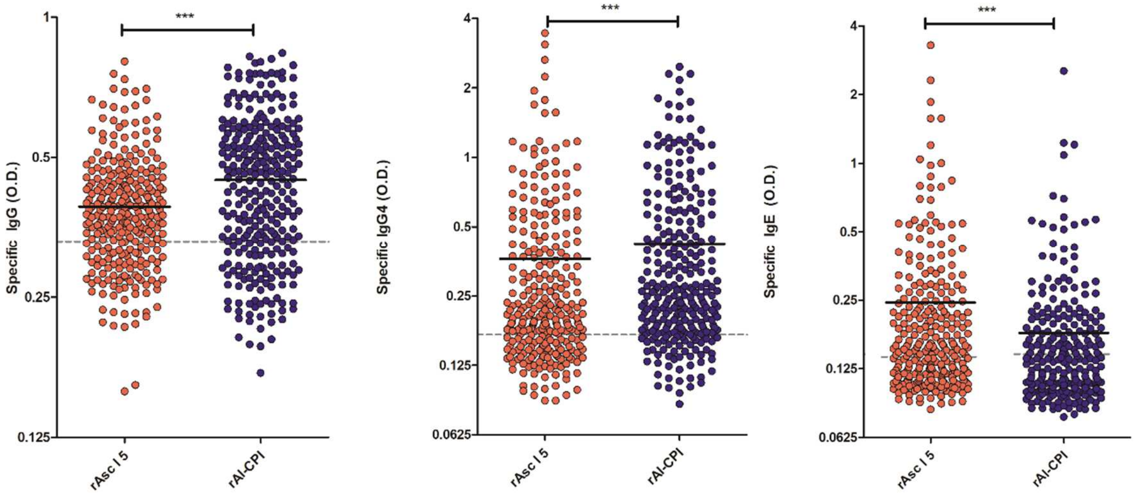

Figure S3. Specific IgE, IgG4 and IgG to Asc l 5 and Al-CPI in the study sample. Each dot represents the mean value obtained for each subject sample (tested by duplicate). The dashed line corresponded to the cut-off value calculated for each assay. *** $p < 0.001$.

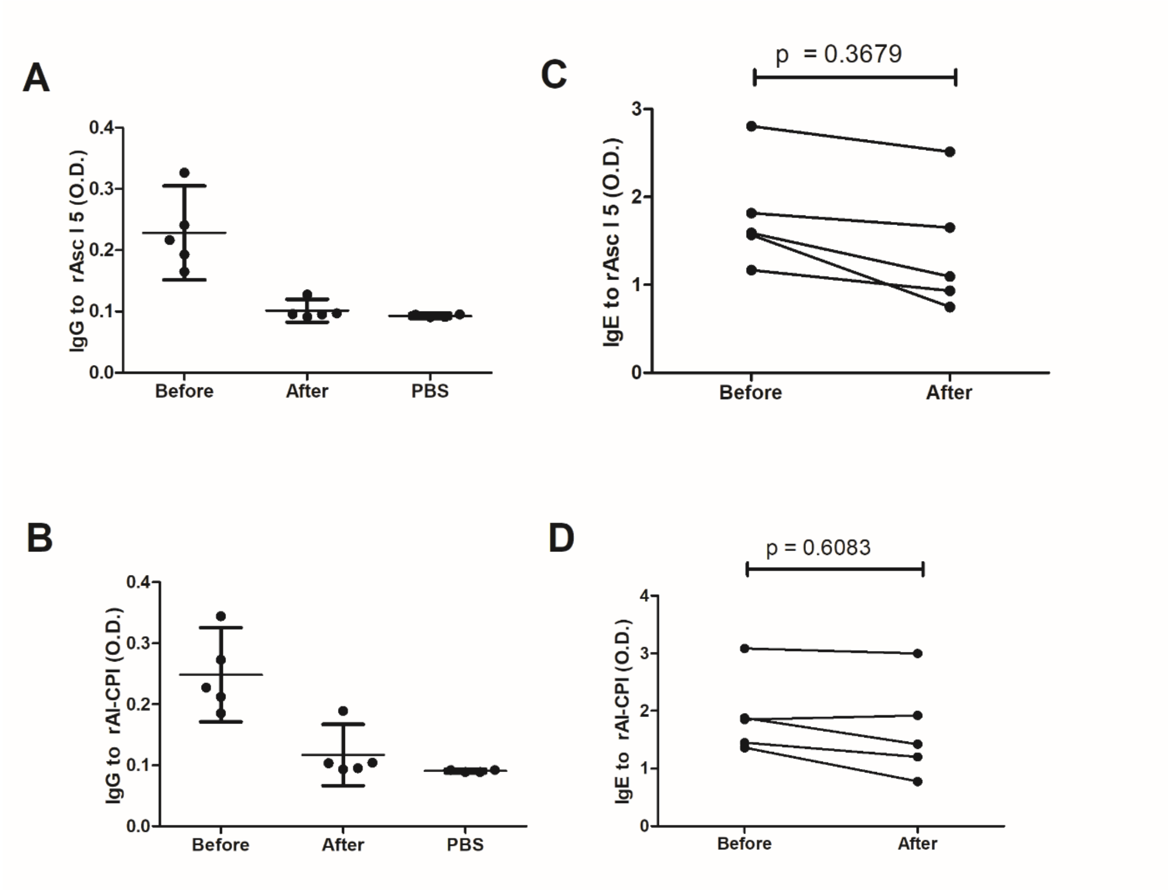

Figure S4. rAsc l 5 or rAl-CPI specific IgG do not block IgE binding to these molecules. IgG levels to rAsc l 5 (A) or rAl-CPI (B) were measured in sera from 5 IgE sensitized individuals before and after incubation with protein G. Thereafter, binding of IgG and IgE to Al-16 or CPI were assessed (C) and (D). Black lines indicate mean values + 95% confidence interval. PBS, buffer control; O.D. optical density.
